# Zoledronic Acid Abrogates Restraint Stress-Induced Macrophage Infiltration, PDGF-AA Expression, and Ovarian Cancer Growth

**DOI:** 10.3390/cancers12092671

**Published:** 2020-09-18

**Authors:** Claudia B. Colon-Echevarria, Tatiana Ortiz, Lizette Maldonado, Melanie J. Hidalgo-Vargas, Jaileene Pérez-Morales, Alexandra N. Aquino-Acevedo, Roberto Herrera-Noriega, Margarita Bonilla-Claudio, Eida M. Castro, Guillermo N. Armaiz-Pena

**Affiliations:** 1 Department of Basic Sciences, Pharmacology Division, School of Medicine, Ponce Health Sciences University, Ponce, PR 00716, USA; ccolon13@stu.psm.edu (C.B.C.-E.); tatianaot@gmail.com (T.O.); limaldonado@psm.edu (L.M.); melanie_hidalgo@outlook.com (M.J.H.-V.); aaquino18@stu.psm.edu (A.N.A.-A.); rherrera18@psm.edu (R.H.-N.); 2 Department of Cancer Epidemiology, H. Lee Moffitt Cancer Center and Research Institute, Tampa, FL 33612, USA; jaileene.perez-morales@moffitt.org; 3 Division of Cancer Biology, Ponce Research Institute, Ponce, PR 00716, USA; mbonilla@psm.edu; 4 Clinical Psychology Program, School of Behavior and Brain Sciences, Ponce Health Sciences University, Ponce, PR 00716, USA; ecastro@psm.edu; 5 Mental Health Division, Ponce Research Institute, Ponce, PR 00716, USA; 6 Division of Women’s Health, Ponce Research Institute, Ponce, PR 00716, USA

**Keywords:** ovarian cancer, adrenergic, macrophages, cytokines, inflammation, *PDGFA*

## Abstract

**Simple Summary:**

Biobehavioral disorders can negatively impact patients with ovarian cancer. Growing evidence suggests that chronic stress can promote tumor progression, the release of inflammatory mediators, and macrophage infiltration into the tumor. However, the role of stress hormones in regulating cancer cell/macrophage crosstalk remains unclear. This study aimed to assess the role of stress hormone-stimulated macrophages in modulating inflammatory networks and ovarian cancer biology. Our data show that stress hormones induced secretion of inflammatory proteins in ovarian cancer cell/macrophage co-cultures. Furthermore, we show that restraint stress leads to cancer growth, macrophage infiltration, and PDGF-AA protein expression in animal models of ovarian cancer. Conversely, zoledronic acid was able to prevent the effects of restraint stress on ovarian cancer growth. Overall, our data suggest a role for stress hormone-stimulated macrophages in ovarian cancer progression and suggest the involvement of PDGF-AA as a key mediator of this process.

**Abstract:**

Multiple studies suggest that chronic stress accelerates the growth of existing tumors by activating the sympathetic nervous system. Data suggest that sustained adrenergic signaling can induce tumor growth, secretion of pro-inflammatory cytokines, and macrophage infiltration. Our goal was to study the role of adrenergic-stimulated macrophages in ovarian cancer biology. Cytokine arrays were used to assess the effect of adrenergic stimulation in pro-tumoral cytokine networks. An orthotopic model of ovarian cancer was used to assess the in vivo effect of daily restraint stress on tumor growth and adrenergic-induced macrophages. Cytokine analyses showed that adrenergic stimulation modulated pro-inflammatory cytokine secretion in a SKOV3ip1 ovarian cancer cell/U937 macrophage co-culture system. Among these, platelet-derived growth factor AA (PDGF-AA), epithelial cell-derived neutrophil-activating peptide (ENA-78), Angiogenin, vascular endothelial growth factor (VEGF), granulocyte-macrophage colony-stimulating factor (GM-CSF), interleukin-5 (IL-5), Lipocalin-2, macrophage migration inhibitory factor (MIF), and transferrin receptor (TfR) were upregulated. Enriched biological processes included cytokine-mediated signaling pathways and positive regulation of cell proliferation. In addition, daily restraint stress increased ovarian cancer growth, infiltration of CD68+ macrophages, and expression of PDGF-AA in orthotopic models of ovarian cancer (SKOV3ip1 and HeyT30), while zoledronic acid, a macrophage-depleting agent, abrogated this effect. Furthermore, in ovarian cancer patients, high *PDGFA* expression correlated with worse outcomes. Here, it is shown that the adrenergic regulation of macrophages and *PDGFA* might play a role in ovarian cancer progression.

## 1. Introduction

Ovarian cancer is the fifth leading cause of cancer death among women in the United States. It also has the highest mortality rate of female reproductive cancers [1,2]. Previous studies have shown that altered psychological states, including chronic stress and depression, contribute to tumor growth and cancer progression [3]. Furthermore, ovarian cancer patients are more likely to suffer from anxiety and depression than the general population [4,5]. This is an important issue to address since psychological stressors contribute to poorer survival and higher mortality of cancer patients [6,7]. Biobehavioral factors have been shown to enhance protumoral factors associated with inflammatory processes [8].

Tumor-associated macrophages (TAMs) are among the most abundant infiltrating immune cells and are key components of the tumor microenvironment. Activated TAMs secrete cytokines, chemokines, proteins, and pro-inflammatory mediators capable of recruiting immune cell populations while ultimately promoting tumor growth [9]. Activation of the sympathetic nervous system and increased levels of catecholamines, epinephrine (Epi), and norepinephrine (NE) in the tumor microenvironment also activate pro-tumoral and pro-inflammatory pathways, induce macrophage infiltration, and promote tumor growth [10]. Several studies have shown that β-adrenergic signaling is capable of mediating the effects of chronic stress on ovarian cancer. This includes activation of metastatic cascades, resistance to chemotherapy, and worse patient outcomes [11,12]. In addition, several studies have shown that adrenergic signaling enhances TAM infiltration, recruitment, and leads to worse survival among ovarian cancer patients [9,13]. Although studies have reported that catecholamines induce TAM infiltration into the tumor microenvironment, the role of stress hormones in the regulation of cancer cells/TAM interaction is not entirely clear.

We investigated the role of adrenergic-stimulated macrophages in modulating pro-inflammatory networks and ovarian cancer biology. This study was guided by the hypothesis that adrenergic signaling induces TAMs to upregulate cytokines involved in pro-inflammatory pathways leading to ovarian cancer progression. We report that alterations in pro-inflammatory networks driven by adrenergic-induced macrophages resulted in enhanced infiltration of TAMs and tumor growth. We demonstrated that the effects of restraint stress on ovarian cancer progression are abrogated by zoledronic acid, a clinically available bisphosphonate, that has been shown to target TAMs in multiple tumor models. Furthermore, our data suggest that adrenergic regulation of macrophages and platelet-derived growth factor AA (PDGF-AA) might play a role in ovarian cancer progression.

## 2. Results

### 2.1. Adrenergic Stimuli Increase Pro-Inflammatory Cytokine Production

**Figure 1:**
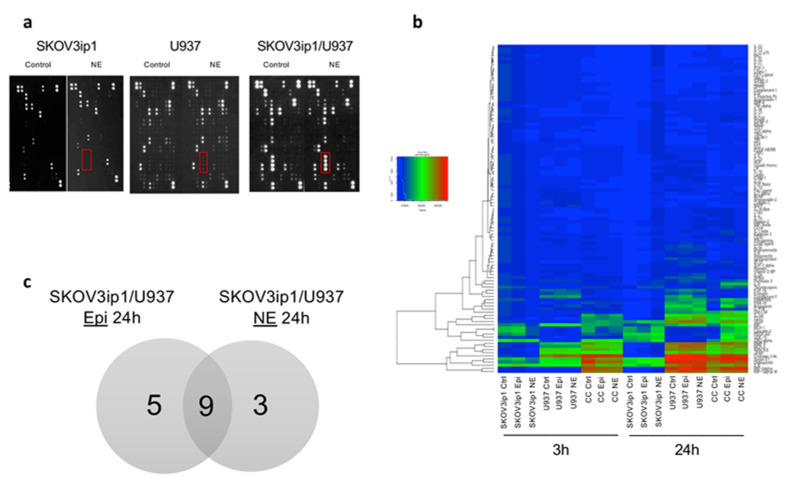
Adrenergic stimuli modulate ovarian cancer cells/macrophage co-cultures cytokine networks. (**a**) Cytokine arrays from SKOV3ip1 and U937 cell lines in monocultures and co-culture conditions 24 h after 10 μM NE exposure. Dots represent cytokines, and red boxes show how changes in cytokine expression are seen in films. Red box demonstrating changes in MIP-1 alpha/beta and MIP-3alpha. (**b**) Heatmap of differentially expressed cytokines between ovarian cancer cells, macrophage monocultures, and co-cultures. The degree of expression is indicated by different colors. Blue, low expression; green, medium expression; red, high expression. Heatmap shows mean raw expression values. (**c**) Venn Diagram of upregulated cytokines shared between epinephrine (Epi)- and norepinephrine (NE)-treated co-culture systems.

To characterize changes in pro-inflammatory networks induced by adrenergic signaling, we analyzed cytokine/chemokine protein expression in supernatants from an ovarian cancer cell/macrophage (SKOV3ip1/U937) co-culture system exposed to epinephrine (Epi) or norepinephrine (NE) (Figure 1a,b). First, SKOV3ip1 monocultures treated with Epi or NE for 3 h (hrs) showed no significant difference in cytokine expression from their respective untreated controls. However, longer exposure of monocultures to stress hormones demonstrated significant changes in cytokines expression compared to untreated controls. SKOV3ip1 monoculture exposure to Epi and NE for 24 h showed significant upregulation in expression, 63, and 95 cytokines, respectively (Table S1).

To understand the influence of macrophages on cancer cell cytokine crosstalk, we evaluated adrenergic-stimulated co-cultures while adjusting for treated monocultures. Short-term (3 h) exposure of SKOV3ip1/U937 co-cultures to Epi and NE resulted in significant upregulation of 12 and 15 cytokines (*p* < 0.05), compared to SKOV3ip1 monoculture conditions. This early response to stress in both conditions resulted in the expression of known macrophage activity modulators such as MCP1, MCP3, macrophage migration inhibitory factor (MIF), uPAR, and matrix metalloprotein 9 (MMP9). Moreover, we observed a more robust cytokine response in SKOV3ip1/U937 co-cultures 24 h after catecholamine exposure. Compared to stimulated SKOV3ip1 monocultures, Epi-treated co-cultures exhibited 45 upregulated cytokines while NE-treated co-cultures had 20 (Table S2). Many of the upregulated cytokines have been shown to be involved in proangiogenic, metastatic, and angiogenic cascades, for example, Chitinase 3-like 1, vascular endothelial growth factor (VEGF), uPAR, RANTES (CCL5), MIP-3alpha, and CD31.

**Figure 2:**
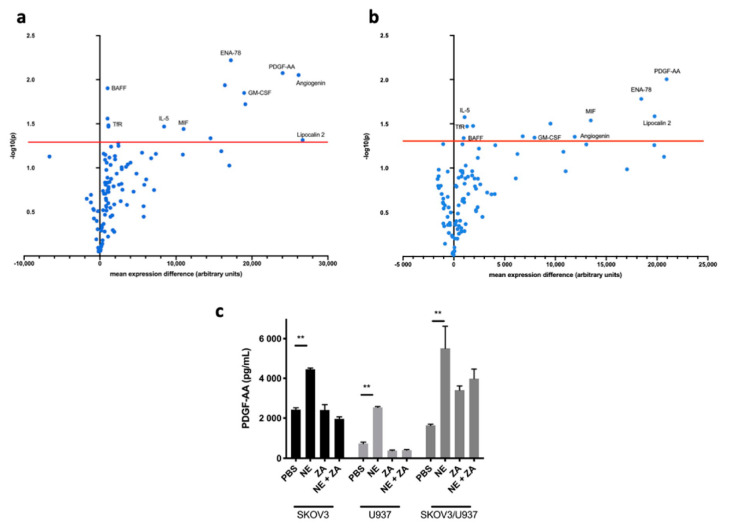
Cytokine profile of adrenergic stimulated ovarian cancer cells/macrophage co-cultures (**a**) Volcano plots showing cytokine changes of SKOV3ip1and U937 co-cultures 24 h after Epi or (**b**) NE treatment. The Y-axis depicts the two-class comparison’s statistical significance in –log notation of the *t*-test’s *p*-value. Thus, the higher the dot, the higher the statistical significance. The X-axis depicts expression compared to monoculture conditions. The farther to the right, the higher the expression. The red line marks the threshold for statistical significance established at *p* < 0.05. Cytokines labeled are shared between Epi and NE groups. (**c**) Zoledronic acid (ZA) prevents NE-induced platelet-derived growth factor AA (PDGF-AA) release in SKOV3 and U937 monocultures and SKOV3/U937 co-cultures. Mean ± SEM. ** *p* < 0.01.

To further characterize the influence of adrenergic stimuli on macrophage-cancer cell crosstalk, we analyzed adrenergic-stimulated co-cultures compared to untreated co-cultures. Co-cultures treated with Epi or NE for 3 h and 24 h exhibited differential expression of pro-inflammatory cytokines compared to untreated co-cultures (Figure 1b). Specifically, at 3 h, neither Epi or NE-treated co-cultures had significantly upregulated cytokines. More importantly, due to the robustness of the effect observed at 24 h, we decided to focus on this time point. Compared to untreated co-cultures, Epi and NE exposure for 24 h differentially upregulated (*p* < 0.05; fold change > 2) 14 and 12 pro-inflammatory cytokines, respectively (Figure 2a,b). Furthermore, nine of these significantly upregulated cytokines overlapped between Epi- and NE-treated groups: PDGF-AA, epithelial cell-derived neutrophil-activating peptide (ENA-78), Lipocalin-2, Angiogenin, interleukin-5 (IL-5), B-cell activating factor (BAFF), MIF, granulocyte-macrophage colony-stimulating factor (GM-CSF), and TfR (Figure 1c). Among these, PDGF-AA and ENA-78 (CXCL5) were found to be the most upregulated in both treatment conditions (Table S3). Furthermore, in order to confirm the role of macrophages in the release of PDGF-AA following adrenergic stimuli, we used Zoledronic acid (ZA) in vitro. ZA, a clinically available bisphosphonate, has demonstrated inhibition of TAMs in culture conditions and multiple tumor models [14,15]. Treatment with 5 μM of ZA blocked NE-induced PDGF-AA release from both SKOV3 cancer cells and U937 differentiated macrophages (Figure 2c).

**Figure 3:**
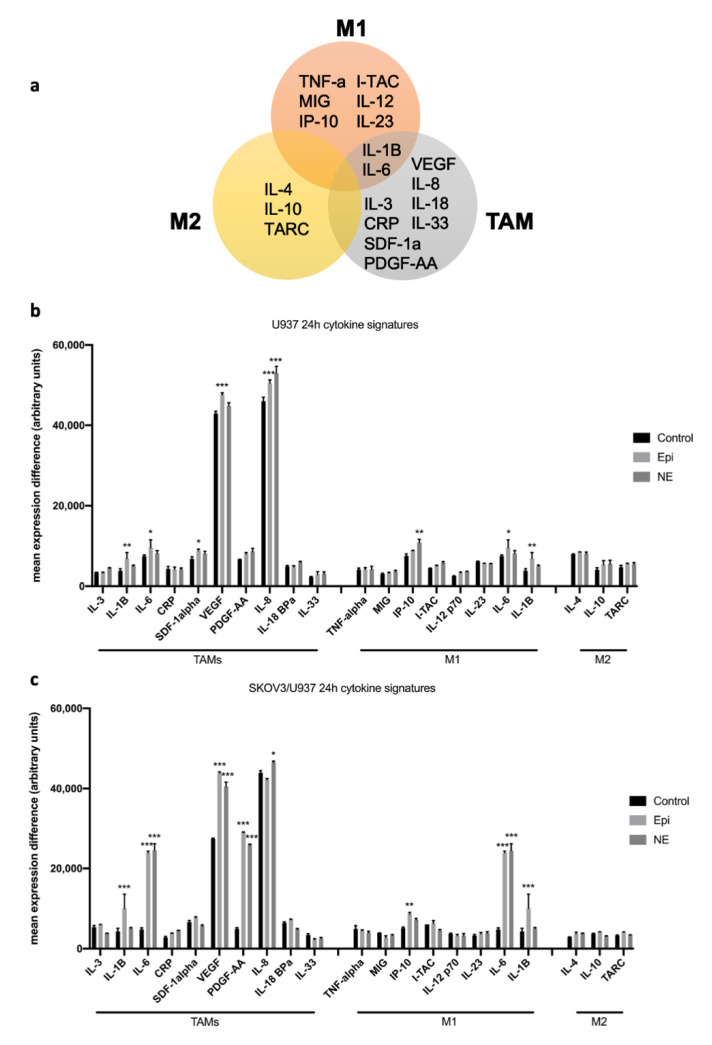
Cytokine signatures associated with Tumor-associated macrophages (TAMs), M1, and M2 phenotypes in U937 monocultures and SKOV3ip1/U937 co-cultures. (**a**) Schematic representation of cytokine signatures categorized by macrophage subsets. (**b**) Cytokine expression signatures in U937 monocultures and (**c**) SKOV3ip1/U937 co-culture conditions after 24-hr exposure to Epi or NE. Mean ± SEM. * *p* < 0.05 ** *p* < 0.01 *** *p* < 0.0001.

In order to understand the potential role of the different subtypes of macrophages under adrenergic stimuli, we analyzed changes in subsets of cytokine signatures in our U937 monoculture and SKOV3ip1/U937 co-culture systems. Here, we utilized existing data from ovarian carcinoma immune profiling studies [16,17,18] to determine if there are changes in cytokine signatures suggestive of M1, M2, and TAMs phenotypes (Figure 3a). The results show that NE-treated SKOV3ip1/U937 co-cultures express significantly more factors from a TAMs signature compared to NE-treated U937 macrophages alone. In addition, these robust changes in cytokine expression profiles were not clearly observed in M1 and M2 signatures. (Figure 3b,c).

### 2.2. Gene Ontology Biological Process and KEGG Pathway Enrichment Analyses of Differentially Expressed Cytokines

**Figure 4:**
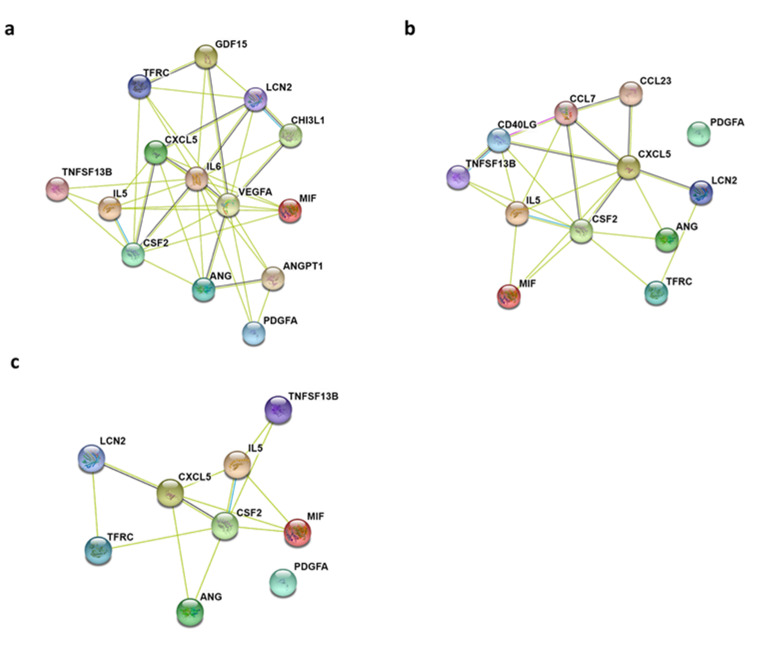
Protein-protein interaction network between differentially expressed cytokines in ovarian cancer–macrophage co-cultures. (**a**) Epi 24 h (**b**) NE 24 h and (**c**) shared cytokines between Epi and NE 24 h treatment. Each node represents a protein, and the edges linking nodes represent known interactions between the two proteins.

In order to understand the biological processes altered by adrenergic stimuli, we constructed protein-protein interaction (PPI) networks from differentially expressed cytokines identified in adrenergic-induced SKOV3ip1/U937 co-cultures. For this purpose, we used the online STRING database (Search Tool for the Retrieval of Interacting Genes/Proteins; string-db.org/), which provides associations and links between query genes and proteins [19]. The results showed a higher number of interactions among cytokines in the Epi-treated network (node degree: 42, clustering coefficient: 0.786) compared to the NE-treated (node degree: 23, clustering coefficient: 0.576) (Table S4). This difference is also evident as the Epi-treated PPI network demonstrates more interactions than the NE-treated and shared cytokine networks (Figure 4).

For Epi-treated co-cultures, the top five significant gene ontology (GO) biological process enriched were positive regulation of protein phosphorylation (GO:0001934), positive regulation of cell population proliferation (GO:0008284), regulation of signaling receptor activity (GO:00010469), regulation of cell population proliferation (GO:0042127), and positive regulation of response to a stimulus (GO:0048584). NE-treated co-cultures were enriched for regulation of signaling receptor activity (GO:00010469), cytokine-mediated signaling pathway (GO:0019221), cellular response to cytokine stimulus (GO:0071345), positive regulation of cell population proliferation (GO:0008284), and immune response (GO:0006955) (Table S4). Among the three networks, two GO biological processes were shared: positive regulation of cell population proliferation (GO:0008284) and regulation of signaling receptor activity (GO:00010469).

Using the STRING platform, we sought to identify signaling pathways that could be modulated through differentially expressed cytokines in adrenergic stimulated co-cultures. KEGG molecular networks provide molecular interactions representing systemic functions in molecular datasets. KEGG pathway enrichment analyses of shared upregulated cytokines between Epi- and NE-treated co-cultures identified 11 signaling networks (Table S5). These include the IL-17 signaling pathway, the Jak-STAT signaling pathway, TNF signaling pathway, and transcriptional misregulation in cancer.

### 2.3. Zoledronic Acid Abrogates Restraint Stress-Induced Macrophage Infiltration, PDGF-AA Expression and Tumor Growth in Orthotopic Mouse Models of Ovarian Cancer

**Figure 5:**
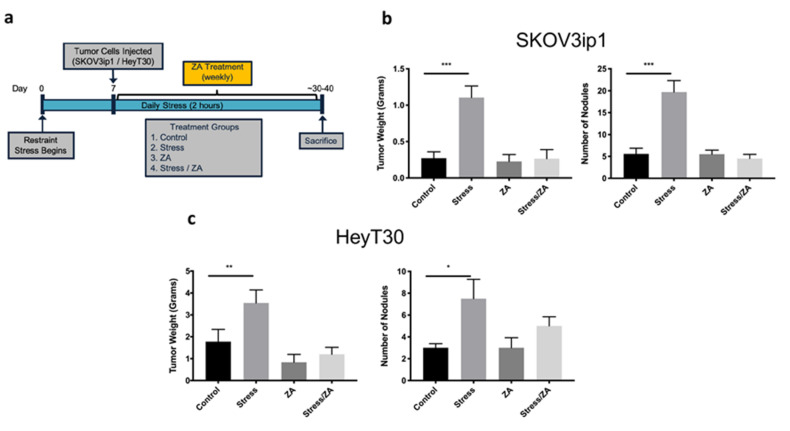
Zoledronic acid (ZA) abrogates restraint stress-induced ovarian cancer tumor growth in an orthotopic Nu/Nu mice model. (**a**) In vivo ovarian cancer orthotopic model timeline (**b**) Tumor weight and tumor nodules in an orthotopic SKOV3ip1 and (**c**) HeyT30 mice model. Stress groups were subjected to daily restraint stress (2 hrs/day), while ZA treatment was administered weekly. Mean ± SEM. * *p* < 0.05 ** *p* < 0.01 *** *p* < 0.0001.

Next, we investigated the role of macrophages in the promotion of ovarian cancer tumor growth in response to sustained adrenergic signaling. Due to the known relationship between stress and TAMs in cancer, we sought to determine if inhibition of TAM activity could abrogate the effects of stress on ovarian cancer progression. For this purpose, the effect of zoledronic acid in restraint stress-induced cancer growth was determined in two ovarian cancer orthotopic mouse models. To induce sympathetic nervous system activation, we utilized an established restraint stress animal model capable of inducing the molecular effects of chronic stress [3]. Mice inoculated intraperitoneally with SKOV3ip1 or HeyT30 ovarian cancer cell lines and randomized into four groups: Control, Stress, ZA, and Stress/ZA. Stress groups were subjected to daily restraint stress (2 hrs/day) and administered either D-PBS or ZA (Figure 5a). In the SKOV3ip1 model, restraint stress increased tumor growth by 4.07-fold (mean difference 0.834 g) and nodule counts by 3.54-fold (mean difference 14.14), while ZA significantly abrogated this effect (Figure 5b; *p* < 0.001). In the HeyT30 model, stress significantly increased tumor growth by 2.0-fold (mean difference 1.769 g), and nodule counts by 2.5-fold (mean difference 4.5). Similarly to the SKOV3ip1 model, ZA treatment prevented the effects of stress on tumor growth and nodule development in the HeyT30 model (Figure 5c; *p* < 0.05).

**Figure 6:**
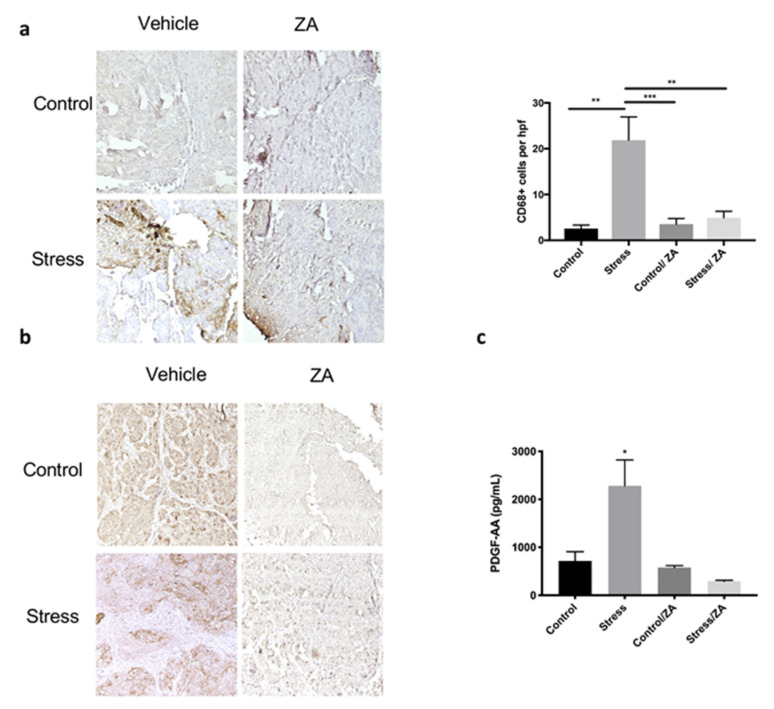
Zoledronic acid (ZA) prevents stress-induced macrophage infiltration and reduces intratumoral PDGF-AA expression in an orthotopic mouse model. Immunohistochemical analysis of SKOV3ip1 tumor samples from mice subjected to restraint stress and ZA showing expression of (**a**) CD68+ and (**b**) PDGF-AA. 20× magnification. (**c**) PDGF-AA protein expression in SKOV3ip1 tumor samples. Mean ± SEM. * *p* < 0.05 ** *p* < 0.01 *** *p* < 0.0001.

After sacrifice, we proceeded to verify macrophage infiltration in formalin-fixed tumor samples from the restraint stress model through CD68 immunohistochemical staining (Figure 6a; *p* < 0.01). Restraint stress increased CD68+ macrophage infiltration into SKOV3ip1 tumors (21.86 CD68+ cells/high-power field (HPF)), and this effect was abrogated by ZA treatment (4.941 CD68+ cells/HPF). Thus, demonstrating ZA was able to prevent the effects of restraint stress by inhibiting macrophage infiltration.

Next, we asked if increased adrenergic signaling enhanced PDGF-AA expression in vivo and if ZA was able to block this effect. The results show that restraint stress significantly increased PDGF-AA protein expression in tumors (stress mean 2283 pg/mL ± 1080 SD), while ZA blocked this effect (stress/ZA mean 293.1 pg/mL ± 45.94 SD) (Figure 6b,c; *p* < 0.01). These data confirm our in vitro findings and suggest a role for adrenergic signaling on PDGF-AA expression.

### 2.4. Elevated Expression of PDGFA Correlates with Poor Outcome in Ovarian Cancer Patients

**Figure 7:**
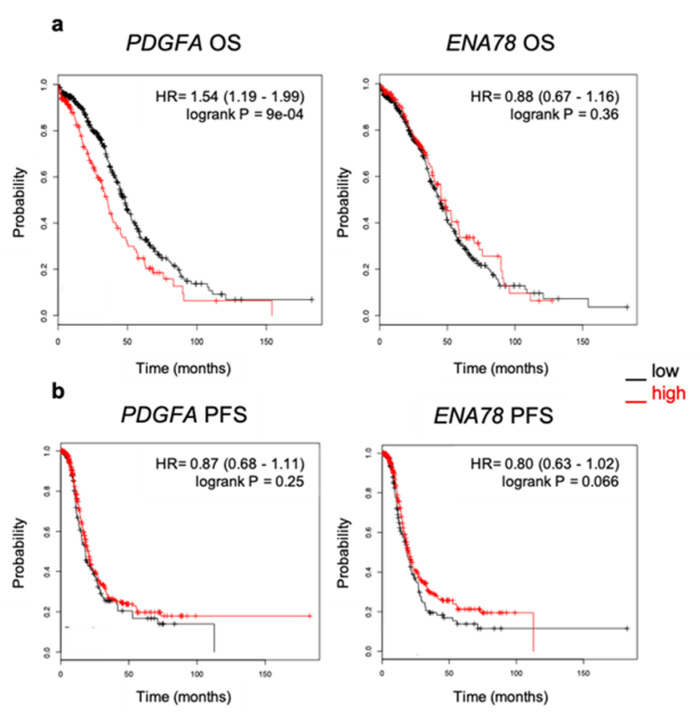
High expression of *PDGFA* correlates with poor outcome in ovarian cancer patients. (**a**) Kaplan–Meier curve of overall survival (OS) (*n* = 557) and (**b**) progression-free survival (PFS) (*n* = 522) in patients with serous epithelial ovarian carcinoma with high or low *PDGFA* and *ENA78* (*CXCL5*) mRNA obtained from KM Plotter using TCGA data. Affymetrix ID: 205463_s_at and 215101_s_at.

Next, we sought to understand the prognostic value of the top two significantly upregulated cytokines identified in our in vitro co-culture system, PDGF-AA and ENA-78. The prognostic value of *PDGFA* and *ENA78* mRNA expression in serous ovarian cancer patients was evaluated using the online tool KM plotter, which evaluates the effect of 22,277 genes and their expression [20]. The analysis was restricted to serious histology within the TCGA dataset (*n* = 565). The results showed that high *PDGFA* (Affymetrix ID: 205463_s_at) expression correlated with decreased overall survival (OS) but did not impact progression-free survival (PFS) in ovarian cancer patients (HR 1.54 (95% CI 1.19–1.99); *p* < 0.001 for OS; HR 0.87 (95% CI 0.68–1.11); *p* < 0.25 for PFS). (Figure 7). In contrast, *ENA78* (Affymetrix ID: 215101_s_at) mRNA expression was not associated with prognostic value.

## 3. Discussion

In this study, we investigated the role of adrenergic signaling in the exacerbation of pro-inflammatory networks in ovarian cancer using a combination of molecular and bioinformatic techniques. Using a co-culture system, we were able to dissect the pro-inflammatory signatures exacerbated through macrophage-cancer cell crosstalk in the tumor microenvironment in the context of chronic stress. The results show that exacerbation of pro-inflammatory cytokines by adrenergic stimuli alters the ovarian cancer microenvironment by inducing activation of protumoral pathways. In addition, we show that pharmacological intervention with ZA is able to prevent the effects of stress on cancer progression by targeting TAMs.

Previous work has shown that ovarian cancer cells can recruit macrophages into the tumor through the secretion of MCP1 [13]. However, the role of these TAMs and their tumor-promoting effects on the tumor microenvironment in the context of stress are not well understood. For the last several years, a growing body of literature has studied the role of inflammation in multiple types of cancers [21,22]. In ovarian cancer, chronic inflammation in the tumor microenvironment has been shown to potentiate tumorigenesis, metastasis, and chemoresistance through cytokine networks capable of immune cell recruitment and stimulating the production of growth factors [23]. For example, macrophage production of IL-6 can induce cell proliferation through the STAT3 axis and production of matrix metalloproteins (MMPs) [24,25]. In addition, previous studies have demonstrated that stress promotes the recruitment of TAMs and the production of inflammatory and angiogenic mediators [26,27].

Since cytokine-mediated communication within the tumor microenvironment is mediated by multiple regulatory networks, cellular stimuli, and cytokine production variations, studying the cytokinome patterns provides an informative network of complex interactions [28]. Thus, understanding interactions within the tumor microenvironment and cytokinome crosstalk and functions will provide insight into the progression of inflammation-driven cancers. This work sought to understand if adrenergic-induced cytokines could modulate the tumor microenvironment and lead to ovarian cancer growth. Dissecting the effects of adrenergic signaling on pro-tumoral macrophage function through exacerbation of cytokine networks will provide new opportunities to target the effects of psychological distress on cancer progression. We identified a pro-inflammatory cytokine network composed of PDGF-AA, ENA-78, GM-CSF, MIF, BAFF, Angiogenin, IL-5, Lipocalin 2, and TfR. Most of which have been involved in promoting angiogenesis and metastasis in various cancers [29,30,31,32]. This inflammatory exacerbation might promote the perpetuation of inflammation in the tumor microenvironment through stimulation of persistent crosstalk between stroma and tumor cells.

Within the identified pro-inflammatory cytokine network signature, cell proliferation was identified as a possible mechanism for adrenergic-induced regulation, since PDGF-AA was strongly upregulated in both catecholamine treated co-cultures. The platelet-derived growth factor (PDGF) signaling has been identified as a major player in cancer progression as mediators of proliferation, angiogenesis, and metastasis [33,34]. The prognostic value of the PDGF family components has been studied in multiple types of cancers [35,36]. Interestingly Bartoschek et al. detected PDGF-AA and PDGF-BB in the majority of tumor samples evaluated, compared with -CC and -DD. More importantly, high levels of PDGF-AA expression were also correlated with poor survival in cervical squamous cell carcinoma, glioblastoma multiforme, and kidney renal clear cell carcinoma [35]. Recent studies have explored the inflammatory expression signatures in ovarian cancers, in which elevated expression of intratumoral *PDGFA* was found to increase the risk of death and correlate with adverse outcomes [18]. In vitro PDGFRα blockade with IMC-3G3, a specific monoclonal antibody, in combination with docetaxel sensitized HeyA8-MDR ovarian cancer cells and induced apoptosis. This effect was also exhibited in tumor-bearing mice after inhibition of tumor growth [37].

The key observation in our study is that ZA was able to prevent the effects of adrenergic signaling on tumor growth and block the infiltration of macrophages. ZA is a bisphosphonate used clinically as an adjuvant cancer treatment and prevention of bone metastasis due to the ability to inhibit osteoclast function and bone resorption [38]. Due to sharing the same lineage as osteoclasts, macrophages uptake ZA resulting in reducing survival, viability, and differentiation [39]. These results demonstrate an alternative pharmacological approach to prevent the effects of stress on tumor progression that vary from targeting components of the adrenergic pathway. Further research is needed to address whether directly targeting the pro-inflammatory cytokine network, led by PDGF-AA and ENA-78, is an effective way to block the effects of adrenergic signaling in vitro. If proven, this approach could be used as therapies for ovarian cancer patients suffering from chronic stress and biobehavioral disorders.

## 4. Materials and Methods

### 4.1. Cell Lines, Treatments, and Co-Culture System

The derivation and source of the established SKOV3, SKOV3ip1, and HeyT30 ovarian cancer cell lines have been reported previously [3,40,41]. SKOV3, SKOV3ip1, HeyT30 ovarian cancer cells, and human U937 monocytic cell line (CRL-1593.2, ATCC, Manassas, VA, USA) were maintained in RPMI 1640 medium, supplemented with 1% antibiotic/antimycotic and 10% fetal bovine serum. All cell lines were grown in 95% O_2_ and 5% CO_2_ at 37 °C, routinely screened for mycoplasma contamination (30-1012k, ATCC). Cell line authentication was performed using short tandem repeat analysis. All experiments were performed with cultures grown at 70–80% confluence.

Cultures were treated with 10 μM epinephrine or norepinephrine in dH_2_O (Sigma-Aldrich, St. Louis, MO, USA) in serum-free media for 3 h and 24 h as necessary. Previous studies have shown 10 μM catecholamine treatment induces oncogenic hallmarks in cancer studies [42,43]. In respective experiments, cultures were pre-treated with 5 μM Zoledronic acid (Sigma-Aldrich, SML0223) diluted in PBS for 48 hrs [15]. Afterward, cultures were treated with 10 μM norepinephrine for 24 h. Control plates were mock-treated with dH_2_O and/or PBS for the same amount of time.

In co-culture experiments, indirect co-culture assays were performed where cancer cells and macrophages were separated by a porous membrane, allowing for the exchange of soluble factors, but no direct physical contact was present. For co-cultures, human U937 cells were differentiated into macrophages by 72 h incubation with 200 nM phorbol 12-myristate 13-acetate (PMA), followed by 24 h incubation in RPMI complete medium. During the 72 h incubation with PMA, U937 monocyte to macrophage differentiation was confirmed by visual analysis of macrophage morphology and attachment to culture plates [44]. Ovarian cancer cells were then seeded (1:1 ratio) on 6-well Transwell inserts with 0.4 μm-pore polycarbonate membranes (Corning, #3412, Glendale, AZ, USA). After a 24 h resting period, cultures were treated with hormones as described previously. After respective treatment, co-culture supernatant was stored at −80 °C for cytokine arrays.

### 4.2. Cytokine Arrays

For cytokine analysis, supernatant from monocultures or co-cultures of ovarian cancer cells/macrophages were used after storage at −80 °C. To measure cytokines, the Proteome Profiler Human XL Cytokine Array kit (ARY022B, R&D Systems, Minneapolis, MN, USA) was used following the manufacturer’s instructions. This array is a membrane-based immunoassay that detects changes in 105 human cytokines simultaneously. After chemiluminescent detection, films were quantified using Quick Spots Tool in Western Vision’s HLImage++ for Array Analysis Software (Version 22.0, Salt Lake City, UT, USA). The average intensity of negative controls per group was subtracted from each cytokine measure to deduct the background. Cytokine expression was normalized to respective monoculture conditions. Raw cytokine expression data are included in Table S6.

To view relative cytokine expression among all treatment conditions, heatmaps were constructed in RStudio (Version 1.0.153, Boston, MA, USA) with the following R packages: RColorBrewer, d3heatmap, and ggplot2 [45,46]. To identify significant changes in cytokine patterns, cytokine expression analysis and volcano plots were constructed using GraphPad Prism (version 8.1.0, San Diego, CA, USA) and Multiexperiment viewer (MeV) program, version 10.2 (Rockville, MD, USA) [47].

### 4.3. Protein-Protein Interaction (PPI) Network Construction

The Search Tool for the Retrieval of Interacting Genes/Proteins (STRING; string-db.org/, Zurich, Switzerland) database provides associations and links between query genes and proteins, creating a PPI network. This network and gene enrichment of differentially expressed cytokines identified previously were constructed with the STRING free online platform (version 11.0) [19]. Network properties include edges: number of interactions; nodes: number of proteins in the network; node degree: average number of interactions; clustering coefficient: the tendency of the network to form clusters. The closer the coefficient is to 1, the more likely the network will form clusters; PPI enrichment *p*-value: statistical significance.

### 4.4. Animal Experiments

For animal studies, 8 to 12-week-old female athymic nude (Nu/Nu) mice were obtained from Taconic Laboratories. All experiments were approved by the Institutional Animal Care and Use Committee at Ponce Health Sciences University (IACUC Protocol #240). Animals were assigned randomly into each group (4 groups of 10 mice): (1) Control (intraperitoneal vehicle injection/once per week), (2) Stress (restraint stress/intraperitoneal vehicle injection/once per week), (3) ZA (1 mg/kg ZA/once per week) and (4) Stress/ZA (restraint stress/1 mg/kg ZA/once per week).

To induce chronic stress, we used a daily physical-restraint-stress model, as previously described [3]. Mice were subjected daily to 2 hrs of restraint-stress. Access to food and water was restricted to all groups during chronic stress protocol. HeyT30 (2.5 × 10^5^ cells/200 µL HBSS, Thermo, Rockford, IL, USA) or SKOV3ip1 (1 × 10^6^ cells/200 µL HBSS) tumor cells were injected intraperitoneally into mice in all groups 7 days after chronic stress protocol began. Mice in experimental groups were treated with 1 mg/kg/once per week ZA until sacrifice. Each group had the following tumor incidence: SKOV3ip1; Control (*n* = 7), Stress (*n* = 7), ZA (*n* = 8), and Stress/ZA (*n* = 8). HeyT30; Control (*n* = 8), Stress (*n* = 8), ZA (*n* = 6), and Stress/ZA (*n* = 9). Animals were sacrificed 30–40 days after tumor cell injection, depending on the tumor burden induced by each cell line. Tumor weight, number of nodules, and nodule location were recorded for each animal immediately after sacrifice. Tumor burden was evaluated by inspecting the peritoneal cavity, removing tumor nodules present, and determining combined tumor weight per mouse. Tumor samples were formalin-fixed and mounted in paraffin blocks for immunohistochemistry analysis. Additional tumor samples were flash-frozen in liquid nitrogen and stored at −80 °C until further processing for ELISA.

### 4.5. Immunohistochemistry

Immunohistochemistry (IHC) was performed on formalin-fixed, paraffin-embedded tumor samples sliced at 4 μm. Samples were stained for CD68 (1:200; MCA1957, BioRad, Hercules, CA, USA), and PDGF-AA (1:250; sc-9974, Santa Cruz Biotechnology, Dallas, TX, USA) following protocol by Pérez-Morales et al., 2018 [48]. Briefly, slides were heated at 65 °C for 30 min and then deparaffinized using graded xylenes. Endogenous peroxidase activity was blocked by incubation in 3% hydrogen peroxide for 15 min. Antigen retrieval was performed using citrate buffer solution for 40 min in a preheated water bath (95–99 °C) and followed by a 30-min cool-down. Protein block solution was added for one hrs at room temperature. Tissues were incubated overnight in the primary antibody at 4–8 °C in a humidity chamber. Protein block, secondary antibody, and 3,3′-Diaminobenzidine (DAB) in the Super Sensitive Link Label IHC kit (LP000-ULE, BioGenex, Fremont, CA, USA) were used for visualization per manufacturer’s instructions. Counterstain was performed using hematoxylin for 20 s, followed by washing with running tap water for five minutes. Slides were dried, mounted, and then blindly scored by two independent investigators. CD68 counts were determined by the amount of CD68+ cells per high power field (HPF) using ImageJ software (Bethesda, MD, USA).

### 4.6. ELISA

Flash-frozen tumor samples were lysed using Bullet Blender Bead Lysis Kit (Next Advance, Pink5E100, Troy, NY, USA). PDGF-AA levels from in vivo tumor lysates were analyzed by ELISA using the Human PDGF-AA ELISA kit (Thermo; EHPDGFA) according to manufacturer’s protocol. Samples were assayed in triplicate, and results represent average mean concentration over triplicate experiments.

### 4.7. Kaplan–Meier Survival Analysis

Kaplan–Meier Plotter Online Tool (KMPlotter; http://kmplot.com, Budapest, Hungary) was used to analyze the relationship between gene expression and ovarian cancer patient clinical outcomes [20]. We investigated overall survival (OS) and progression-free survival (PFS) in patients with high and low expression of *PDGFA* and *ENA78* (*CXCL5*) (Affymetrix IDs: 205463_s_at and 215101_s_at, Thermo). The analysis was restricted to ovarian cancer serous subtype and TCGA database. Hazard ratio (HR) with 95% confidence interval (CI) and log-rank *p*-value were retrieved from the online analysis. The threshold for significance was established at *p* < 0.05. HR = 1 indicates that there is no difference in survival between the two groups. HR > 1 or < 1 indicates that one of the groups had better survival than the other.

### 4.8. Statistical Analysis

Statistical analyses were performed using GraphPad Prism (version 8.1.0). Cytokine expression analysis was performed using raw expression values (arbitrary units). The student’s *t*-test with Welch’s approximation for unequal variances was performed to identify significant differences in cytokine expression between groups. One-way ANOVA with Dunnett’s multiple comparison test was performed to analyze differences in tumor weight and nodules, CD68, and PDGF-AA expression in tumors. Two-way ANOVA with Dunnett’s multiple comparison test was performed for PDGF-AA expression in vitro and macrophage signature expression analysis. The log-rank test was obtained from KM Plotter. The statistical significance threshold was established at *p* < 0.05.

## 5. Conclusions

In conclusion, we demonstrated that adrenergic signaling is able to exacerbate TAM infiltration, and promote a pro-inflammatory tumor microenvironment, while ZA abrogated this effect. These results provide new insights into the pro-inflammatory profile of the tumor microenvironment and the role of adrenergic signaling in ovarian cancer. Further studies are needed to fully understand the signaling cascades activated by the inflammatory cytokines exacerbated by adrenergic signaling and how ZA affects them. In addition, cytokine profiling provides new opportunities to identify potential biomarkers for early detection and understanding of the damaging effects of behavioral stress on tumoral profiles.

## Supplementary Materials

The following are available online at https://www.mdpi.com/2072-6694/12/9/2671/s1, Table S1: Significantly upregulated cytokines in 24 h Epi- or NE-treated SKOV3ip1 monocultures (adjusted for untreated monocultures), Table S2: Significantly upregulated cytokines in 24 h Epi- or NE-treated co-cultures (adjusted for treated monocultures), Table S3: Significantly upregulated cytokines in 24 h Epi- or NE-treated ovarian cancer cell/macrophage co-cultures, Table S4: Top five gene ontology biological processes enriched from differentially regulated cytokines after adrenergic stimuli, Table S5: KEGG pathway analysis of enriched signaling networks from differentially regulated cytokines after adrenergic stimuli, Table S6: Raw cytokine expression data for monoculture and co-cultures.

## Abbreviations

DAB	3,3′-Diaminobenzidine
Epi	epinephrine
NE	norepinephrine
ZA	zoledronic acid
hrs	hours
BAFF	B-cell activating factor
ENA-78	epithelial cell-derived neutrophil-activating peptide
GM-CSF	granulocyte-macrophage colony-stimulating factor
GO	gene ontology
HR	hazard ratio
HPF	high-power field
IL	interleukin
MIF	macrophage migration inhibitory factor
OS	overall survival
PDGF-AA	platelet-derived growth factor AA
PFS	progression-free survival
TAMs	tumor-associated macrophages
TfR	transferrin receptor
VEGF	vascular endothelial growth factor
PPI	protein-protein interaction
KEGG	Kyoto Encyclopedia of Genes and Genomes
TNF	tumor necrosis factor
STRING	Search Tool for the Retrieval of Interacting Genes/Proteins
KM	Kaplan–Meier
MMP	matrix metalloprotein
PMA	phorbol 12-myristate 13-acetate
MeV	Multiexperiment viewer
IHC	immunohistochemistry
CI	confidence interval
